# AnnotateGenomicRegions: a web application

**DOI:** 10.1186/1471-2105-15-S1-S8

**Published:** 2014-01-10

**Authors:** Luca Zammataro, Rita DeMolfetta, Gabriele Bucci, Arnaud Ceol, Heiko Muller

**Affiliations:** 1 Computational Research, Center for Genomic Science of IIT@SEMM, Istituto Italiano di Tecnologia (IIT), Via Adamello 16, 20139 Milan, Italy; 2 European School of Molecular Medicine (SEMM), Via Adamello 16, 20139 Milan, Italy

## Abstract

**Background:**

Modern genomic technologies produce large amounts of data that can be mapped to specific regions in the genome. Among the first steps in interpreting the results is annotation of genomic regions with known features such as genes, promoters, CpG islands etc. Several tools have been published to perform this task. However, using these tools often requires a significant amount of bioinformatics skills and/or downloading and installing dedicated software.

**Results:**

Here we present AnnotateGenomicRegions, a web application that accepts genomic regions as input and outputs a selection of overlapping and/or neighboring genome annotations. Supported organisms include human (hg18, hg19), mouse (mm8, mm9, mm10), zebrafish (danRer7), and Saccharomyces cerevisiae (sacCer2, sacCer3). AnnotateGenomicRegions is accessible online on a public server or can be installed locally. Some frequently used annotations and genomes are embedded in the application while custom annotations may be added by the user.

**Conclusions:**

The increasing spread of genomic technologies generates the need for a simple-to-use annotation tool for genomic regions that can be used by biologists and bioinformaticians alike. AnnotateGenomicRegions meets this demand. AnnotateGenomicRegions is an open-source web application that can be installed on any personal computer or institute server. AnnotateGenomicRegions is available at: http://cru.genomics.iit.it/AnnotateGenomicRegions.

## Background

A common denominator for all applications of Next Generation Sequencing technology is the need to annotate genomic regions of interest. This task is usually performed by bioinformaticians who prepare the data as custom tracks for genome browsers and use a set of additional tools to produce tabular annotations to be scrutinized by biologists. Using these tools often requires a significant amount of bioinformatics skills and/or downloading and installing dedicated software. Tools have been developed that comprise functional annotation, for example CisGenome, W-ChIPeaks, Sole-Search, or CASSys [1-4]. These tools focus on the identification of enriched regions in chromatin immunoprecipitation sequencing (ChIP-seq) experiments and annotation of genomic regions is provided as a side-aspect. Therefore, using these tools for annotation purposes only is cumbersome. Command-line tools such as BEDTools [5] are very powerful at identifying overlapping regions in two files provided in browser extensible data (BED) format. Details on this format can be found at https://genome.ucsc.edu/FAQ/FAQformat.html. But being command-line tools, they are hard to use for biologists. The same is true for the BioConductor ChIPpeakAnno package [6]. Tools such as the EnsEMBL Ruby API [7] require considerable programming skills, which precludes widespread use by biologists.

Galaxy [8] is a sophisticated web-based suite of genome analysis tools that can also perform annotation of genomic regions as part of the "Operate on Genomic Intervals" menu option. It is an expert tool that requires some familiarity. The option "Fetch closest non-overlapping feature" will find annotations that have been defined as "neighbors" in this work. The file defining the neighbors must be uploaded along with the query regions. No default annotations for neighbor fetching are provided. Only one annotation can be fetched at the time. Identification of overlapping features requires the use of a different menu option ("Intersect"). The UCSC table browser [9] has the functionalities required to annotate sets of genomic regions. However, the input is restricted to 1,000 regions, which makes this tool cumbersome to use for the annotation of large genomic experiments, for example.

A widely accepted, web-based annotation tool available to bioinformaticians and biologists with widely varying skill levels is not available. Here we present AnnotateGenomicRegions [10], a web application that accepts genomic regions as input and outputs overlapping and/or neighboring genome annotations chosen on a simple web-form.

## Implementation

AnnotateGenomicRegions has been developed using Java Enterprise technology on the NetBeans 7.1 Integrated Development Environment http://netbeans.org/ and runs on a Glassfish version 3.1 web server http://glassfish.java.net/. We also successfully tested other Java Enterprise Edition servers such as Apache TomEE 1.5.2 http://tomee.apache.org, JBOSS community edition 6.1 http://www.jboss.org/, and WebSphere community edition 3.0 http://www-03.ibm.com/software/products/us/en/appserv-wasce/. Java Server Faces https://javaserverfaces.java.net/ and PrimeFaces http://primefaces.org/ frameworks have been chosen for rendering the graphical user interface. Apache Maven http://maven.apache.org/ is used as a software management tool. AnnotateGenomicRegions relies on a set of Java beans to process the annotation queries and returns the annotations as zipped, tab-delimited tables. A set of tutorials and examples are provided to allow the user to get started quickly. The annotations are kept on the server for two hours before being deleted. AnnotateGenomicRegions is a Sourceforge project and can be downloaded from http://sourceforge.net/projects/annotatelocus/ along with detailed descriptions of input and output formats.

AnnotateGenomicRegions provides the annotations and genomes most frequently requested by biologists working with the developers of the tool. Currently, the annotations comprise Refseq transcripts [11], EnsEMBL transcripts [12], all_mrna transcripts [9], CpG islands, and promoter regions of transcripts. Promoter regions are defined as 1 kb regions upstream and downstream of the corresponding transcription start site. The annotations are downloaded from the UCSC genome browser [13], formatted, sorted by chromosome, start position and end position, and incorporated in the annotation pipeline. A comprehensive list of annotations and genomes for the release of October 2012 (Oct2012) is shown in Additional file 1. The users are welcome to request additional annotations or genomes that we may incorporate in the online application using the "Contact" form. Annotations will be updated on a yearly basis. AnnotateGenomicRegions doesn't strive to provide a comprehensive list of annotations for all available genome assemblies. AnnotateGenomicRegions permits uploading customized annotations instead. Furthermore, researchers necessitating annotations not included in AnnotateGenomicRegions are encouraged to run their own local and customized installation.

## Results and discussion

Conscious of the need for an easy to use application for annotating genomic regions, we have developed and made available online AnnotateGenomicRegions, a fast web application that allows submitting a list of genomic regions and displays the corresponding annotations in a web page that can be exported in a tab delimited format recognized by spreadsheet programs.

**Figure 1:**
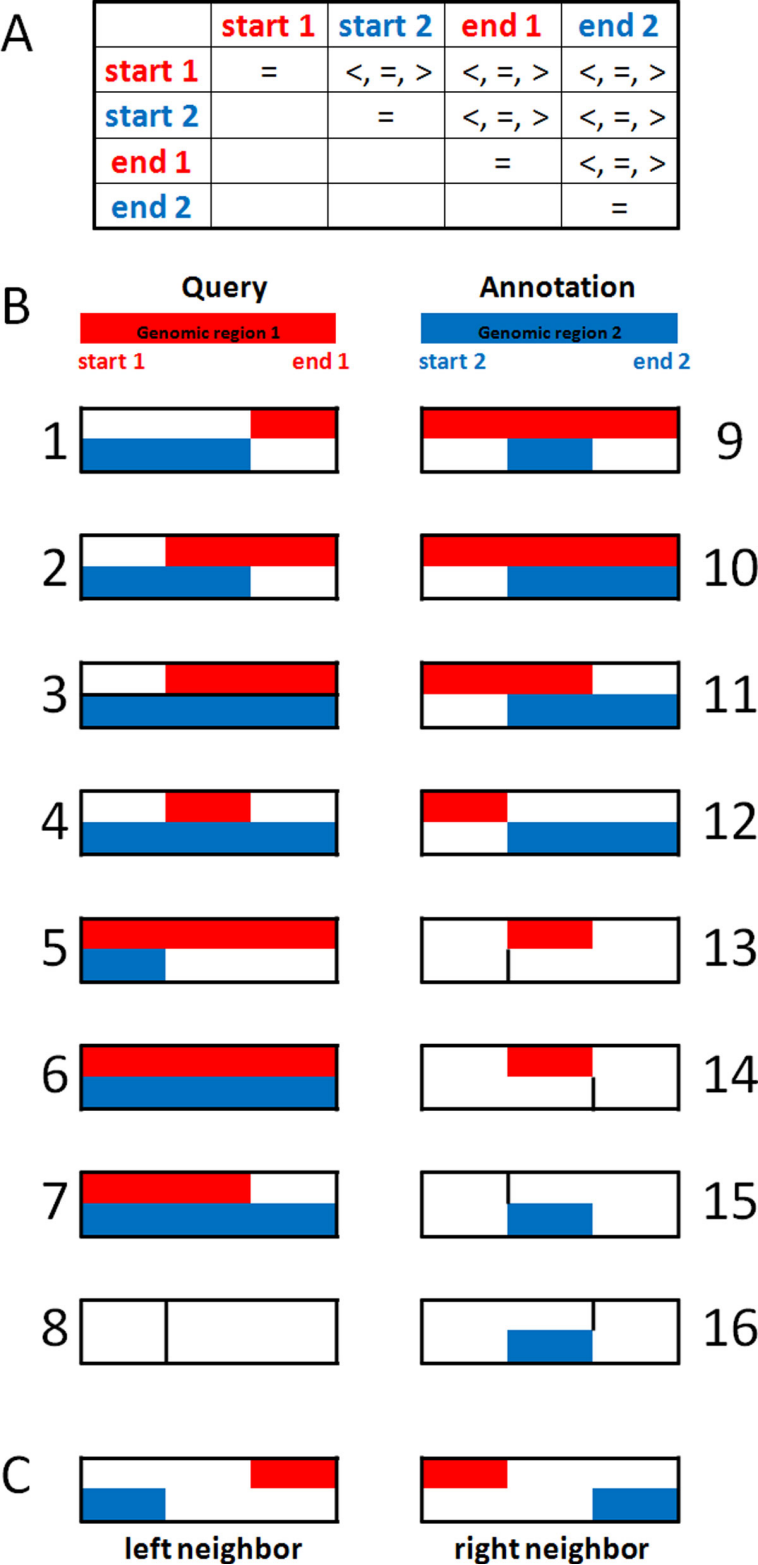
**Overlap and neighbor queries**. A) For two genomic regions located on the same chromosome, the possible relations (>, =, <) between start and end positions of the elements are listed. Note that not all configurations are realistic, e.g. start x > end x is forbidden by definition. B) Comprehensive overview of all overlap types between queries (red bars) and annotations (blue bars) are shown, including overlaps of genomic elements of size zero (cases 8, 13, 14, 15, 16). C) Neighbor annotations are defined as the closest annotation to the query that is not overlapping the query in the sense defined by panel B. Left neighbors are located upstream and right neighbors are located downstream of the query element. For left neighbors, the distance is measured between query start and annotation end. For right neighbors, the distance is measured between query end and annotation start.

AnnotateGenomicRegions annotates sets of genomic regions of interest with overlapping and/or neighboring features that are mapped to the genome. Genomic regions of interest can be derived from experiments such as ChIP-seq, DNase I hypersensitive sites sequencing (DNase-seq), methylation profiling using reduced representation bisulfite sequencing (Methyl RRBS), quantitation of small RNAs by massively parallel sequencing (Small RNA-seq), resequencing etc., or might be derived from in-silico screens such as regions harboring a given DNA-motif. A question that typically needs to be answered early in the analysis regards the relation of the experimentally defined regions with known features in the genome. User provided and embedded annotations are annotated for overlapping and/or neighboring annotations by AnnotateGenomicRegions. The definitions of overlaps and neighborhood of genomic regions are described in Figure 1.

We consider two genomic regions in Figure 1: One region represents a query (shown in red) and the other region represents an annotation (shown in blue). Both regions have a start and an end position. Figure 1A lists the possible relations between the start and the end positions of the two genomic regions. We distinguish three possible relations between any two positions: larger than, equal to, and smaller than. From these relations follow all types of overlaps that can be observed during the annotation process, which are shown in Figure 1B. There are 16 possible types of overlaps between the query and the annotation regions, including overlaps of regions with length zero. Such regions are often found in data sets on genome variation and might describe insertion points, for example. Figure 1C depicts what is intended as neighboring annotations in AnnotateGenomicRegions. Neighboring annotations are not overlapping the query region as defined in Figure 1B and are closest to the query region. Here, "closest" doesn't relate to the physical distance on the chromosome. It only means that there is no other genomic region in between the query region and the neighboring annotation.

**Figure 2:**
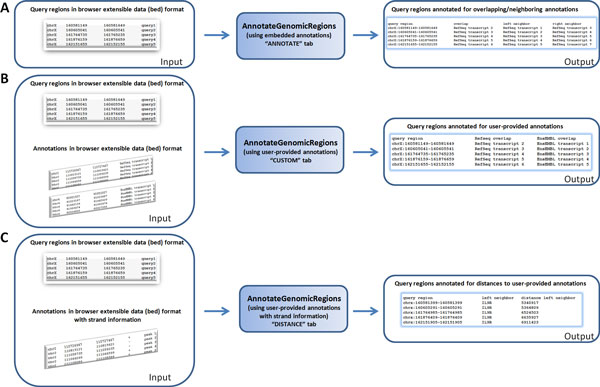
**Use cases**. The three use cases are shown schematically. In all cases, the output can be downloaded and pasted into a spreadsheet. A) User provides query regions and selects desired annotations embedded in AnnotateGenomicRegions. Output lists overlapping and/or neighboring annotations as defined by the user. B) User provides query regions and annotations. Output lists overlapping and/or neighboring annotations as defined by the user. C) User provides query regions and annotations with strand information. Output lists neighboring annotations and corresponding distance.

AnnotateGenomicRegions has been designed to satisfy three use-cases that are shown in Figure 2. The use-cases differ slightly in the type of input that is required from the user. In the easiest case, the user submits a set of genomic regions and annotates them for annotations that are embedded in AnnotateGenomicRegions (Figure 2A). Naturally, this mode of using the tool is limited to the embedded genome annotations. The output is a table that lists overlapping and/or neighboring annotations as chosen by the user for each query region.

In cases where the embedded annotations are not sufficient, the user may upload his/her own annotations. The uploaded files must be in BED format. As shown in Figure 2B, in this scenario the user has to upload both the query regions and all of the required annotation files. The output is represented by a table that annotates the query regions for overlaps/neighborhood of each of the uploaded annotation files.

Sometimes the user may wish to know the distance between a query region and a neighboring annotation. This often happens when transcription factors are studied and the distance to the nearest transcription start site is of interest. Figure 2C shows the input that the user must provide for obtaining distance annotations. The only difference from the use-case described in Figure 2B is that the uploaded annotation files must contain strand information. The output then lists the name of the annotation and the corresponding distance. Note that the strand information is required because distances of interest often include the 5' end or the 3' end of an annotation. By convention, the 5' end is identical to the start base of an annotation on the plus strand and the end base of the annotation on the minus strand (vice versa for 3' ends).

**Figure 3:**
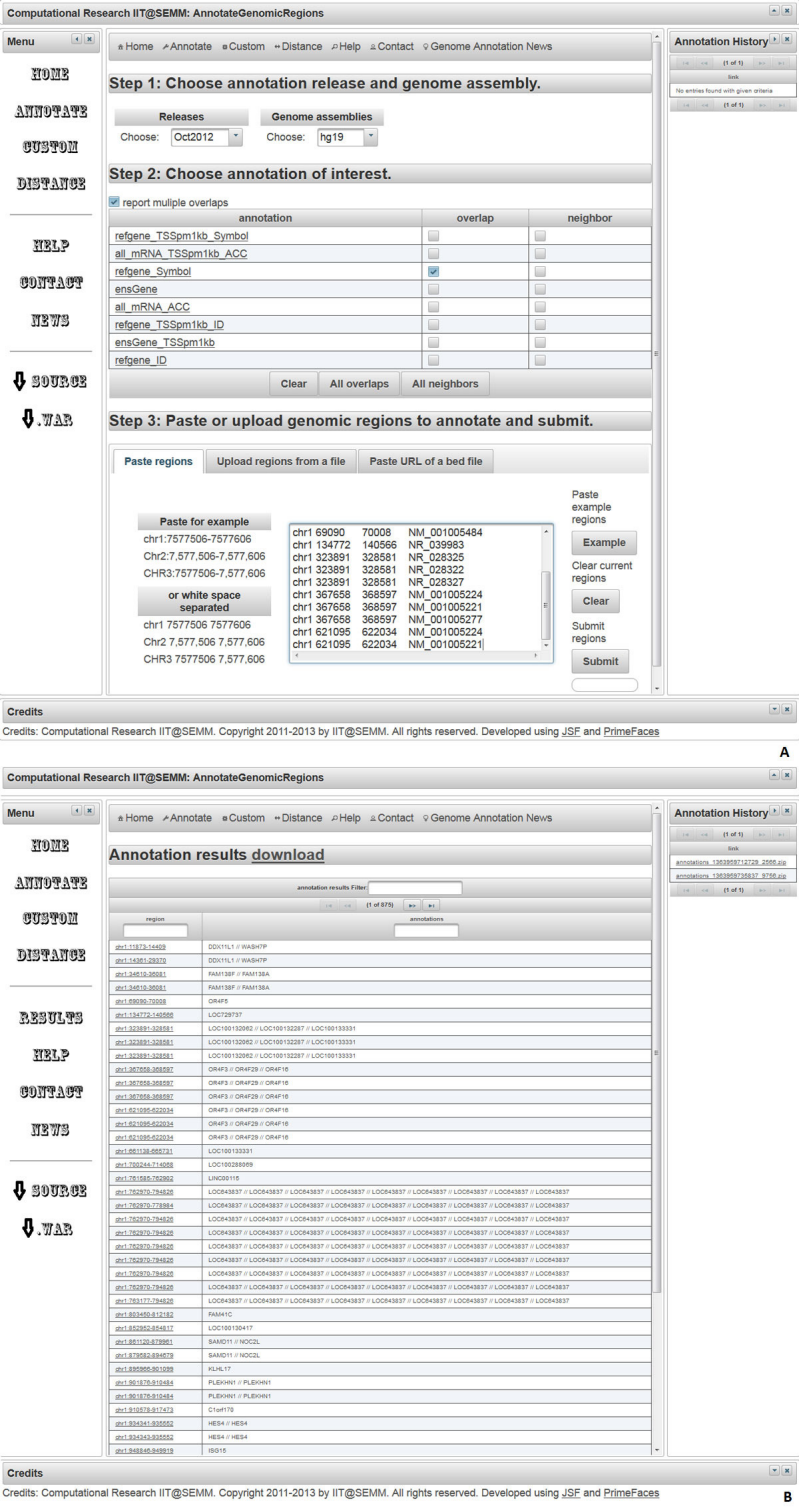
**Screenshots of AnnotateGenomicRegions**. A) Annotate pane. Here, the user uploads query regions, selects desired release, the genome and embedded annotations of interest, defines if multiple hits shall be displayed, and submits the query. Query regions can be uploaded by pasting them directly, by uploading a file, or by providing a hyperlink to a file in the appropriate format. The format of the query regions can be any of the following position coordinates such as chr1:7577506-7577606 or Chr2:7,577,506-7,577,606 or CHR3:7577506-7,577,606 (case insensitive, with and without thousands separators, colon between chr and start, minus between start and end). Colon and minus characters are not compulsory and space or tabulator characters can be used instead (also called white space characters). Allowing tabulator characters as separators permits pasting query regions directly from a spreadsheet holding chromosome, start, and end positions in separate columns. Note that query regions can be duplicated. These duplications represent alternatively spliced transcripts with identical start and stop positions but with different transcript identifiers. Duplicated regions will be annotated independently from each other as if they were not duplicated. B) Output example. The output lists all the annotations found for each query region. In this example, all RefSeq Symbols for transcription units overlapping the query regions are shown, separated by two forward slash ("/") characters. A hyperlink for downloading the latest results is shown. The hyperlinks corresponding to queries executed earlier are displayed in the Annotation history. Each query region is hyperlinked for display in the UCSC genome browser. Duplicated query regions are listed independently from each other and will display identical annotations. Keeping the duplicates helps keeping the output compatible with the input so that the output can be pasted easily side-by-side to annotations obtained previously. Note that the results page contains a filter function ("annotation results filter"). Typing in the associated text field will filter out all rows that do not contain the typed word in any column. This feature is useful if a researcher is interested in a given gene, for example. Filters can be applied separately to each column by typing in the text fields labeled as "region" or "annotations".

Figure 3 shows screenshots of AnnotateGenomicRegions. On the "**Annotate**" pane (Figure 3A) the user is invited to choose the annotation release, the genome, the desired features for annotation, and whether the annotations of the neighboring regions should be accepted. The query regions should be pasted or uploaded in BED format or position coordinates (chromosome:start-end). Upon submitting the query, the results are displayed in tabular form (Figure 3B), can be downloaded in zip format, and pasted into a spreadsheet program such as Microsoft Excel or LibreOffice. A hyperlink allows displaying each region in the UCSC genome browser [13].

For non-standard annotations, a "**CUSTOM**" menu option has been provided. Here, the user can upload an annotation file in BED format along with the queries. The user chooses the number of desired annotation files, browses to the local files containing the annotations, specifies the column indices for chromosome, start, end, and annotation name, and chooses whether overlap or neighbors queries are desired. When submitting the queries, the annotations will be uploaded to the server, processed for fast annotation, and the annotations will be provided as a zipped output file. When the correct genome assembly is chosen on the web form, the regions may be viewed in the UCSC genome browser.

Finally, it is possible to calculate the distance between a query region and a given genome annotation. This task can be accomplished using the "**DISTANCE**" menu option. The annotations used for distance calculations must be provided by the user in the format:

$$\begin{array}{ccccc} \text{"Chromosome} & \text{start} & \text{end} & \text{strand} & \text{annotation"} \end{array}$$

The user can calculate the distance to the 5' end, the 3' end, the start, the end, or the center position of the annotation. The distance is calculated either to the start, the end, or the center position of the query region, as defined by the user.

**Figure 4:**
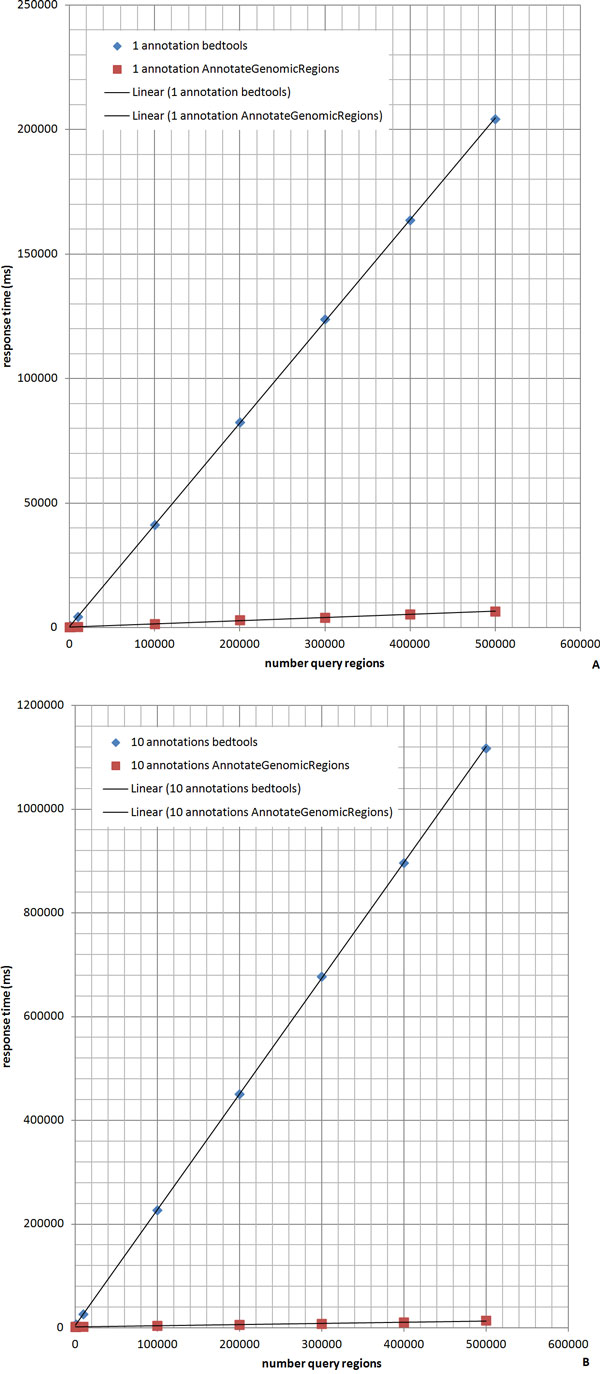
**Comparison of AnnotateGenomicRegions and BEDTools**. AnnotateGenomicRegions and BEDTools were used to annotate identical query regions with identical annotation files. The number of query regions was varied between 10 and 500,000. The response times for AnnotateGenomicRegions were found to be two times faster for up to 100 query regions. For 500,000 query regions, AnnotateGenomicRegions was found to be 30 times faster for 1 annotation (A) and 80 times faster for 10 annotations (B). The tests were performed on an Ubuntu Server Linux computer with 12 CPU's and 16 GB RAM.

The speed of the annotation process using AnnotateGenomicRegions was compared to the speed of BEDTools [5]. BEDTools is popular in the bioinformatics community and provides command line functionality that permits annotating a set of query regions with one or more annotation files. BEDTools uses C++ libraries for annotation that were originally developed for the UCSC genome browser [5,13]. To speed up the annotation process, these libraries employ a binning scheme to the genomic regions of the annotations and build an index on the bins obtained. AnnotateGenomicRegions, on the other hand, uses hash tables for each chromosome holding the annotations sorted by start position. Auxiliary hash tables are then used that memorize the position of the last annotation found to be overlapping or neighboring a query region. Since the query regions are sorted by start position, the auxiliary hash tables help minimizing the number of times a given annotation is visited. Figure 4 shows the response times of AnnotateGenomicRegions and of BEDTools when used for annotating between 10 and 500,000 query regions with one or ten different annotations. It can be seen that AnnotateGenomicRegions is significantly faster than BEDTools, particularly for large numbers of query regions. The response times for up to 300,000 query regions remain below 10 seconds. This amount of query regions exceeds the number of regions to be annotated in a typical genomic experiment several fold.

## Conclusions

AnnotateGenomicRegions is a web-application that allows researchers with a wide range of bioinformatics skills to annotate genomic regions of interest, e.g. ChIP-seq peaks, with overlapping or neighboring annotations. In contrast to other tools, AnnotateGenomicRegions, can be used easily by non-experts. The annotations are provided as tab-delimited text files that can be pasted into a spreadsheet. Query regions are hyperlinked for viewing them in the UCSC Genome Browser. Commonly used annotations such as Refseq transcripts [11], EnsEMBL transcripts [12], or CpG islands are downloaded regularly from the UCSC genome browser repository and made available for instant annotation of genomic regions of interest. Users are invited to leave their feedback using the contact form to improve the software or to participate in future developments of the tool.

## Abbreviations

BED: browser extensible data; ChIP-seq: Chromatin Immunoprecipiation followed by massively parallel sequencing; danRer7: July 2010 zebrafish (Danio rerio) Zv9 assembly produced by The Wellcome Trust Sanger Institute; DNase-seq: DNase I hypersensitive sites sequencing; hg18: March 2006 human reference sequence (NCBI Build 36.1); hg19: February 2009 human reference sequence (Genome Reference Consortium Human Build 37); Methyl-RRBS: genome-wide methylation profiling using reduced representation bisulfite sequencing; mm10: December 2011 Mus musculus assembly (Genome Reference Consortium Mouse Build 38); mm8: February 2006 Mus musculus assembly (Genome Reference Consortium Mouse Build 36); mm9: July 2007 Mus musculus assembly (Genome Reference Consortium Mouse Build 37); Oct2012: October 2012 release of AnnotateGenomicRegions embedded annotations; sacCer2: June 2008 Saccharomyces cerevisiae genome assembly based on sequence in the Saccharomyces Genome Database; SacCer3: April 2011 Saccharomyces cerevisiae genome assembly based on sequence in the Saccharomyces Genome Database; smallRNA-seq: quantitation of small RNAs by massively parallel sequencing.

## Availability and requirements

•Project name: AnnotateGenomicRegions

•Project home page: http://cru.genomics.iit.it/AnnotateGenomicRegions

•Operating system(s): Platform independent

•Programming language: Java

•Other requirements: Java 1.6 or higher, Glassfish 3.1 or higher

•License: Apache license

•Any restrictions to use by non-academics: no restrictions

## Competing interests

The authors declare that they have no competing interests.

## Authors' contributions

LZ provided, formats, and maintains the annotations. RD provided the file uploads module. GB took care of application deployment and maintenance. AC maintains the project code. HM conceived the project, provided the initial implementation, and wrote the manuscript. All authors contributed to the editing of the manuscript. All authors read and approved the final manuscript.

## Supplementary Material

Additional file 1 **Annotations and genomes provided by AnnotateGenomicRegions**. The annotation name column shows the file name that is displayed by the web application. The UCSC download file name lists the name of the file at the UCSC genome browser repository that is used as the annotation source. hg19, hg18, mm10, mm9, mm8, danRer7, sacCer3, and sacCer2 denote the genome assemblies for which annotations are embedded in the web application. The region description column holds a short description of the annotation file content. The region name example shows examples of the names associated with each genomic region. Click here for file

## Declarations

This publication was funded by the Italian Institute of Technology (IIT), Genoa, Italy.

This article has been published as part of *BMC Bioinformatics *Volume 15 Supplement 1, 2014: Integrated Bio-Search: Selected Works from the 12th International Workshop on Network Tools and Applications in Biology (NETTAB 2012). The full contents of the supplement are available online at http://www.biomedcentral.com/bmcbioinformatics/supplements/15/S1.

## References

[B1] Blahnik KR Dou L O'Geen H McPhillips T Xu X Cao AR Iyengar S Nicolet CM Ludascher B Korf I Farnham PJ Sole-Search: an integrated analysis program for peak detection and functional annotation using ChIP-seq data Nucleic Acids Res 2010 38 3 e13 19906703 10.1093/nar/gkp1012 PMC2817454

[B2] Lan X Bonneville R Apostolos J Wu W Jin VX W-ChIPeaks: a comprehensive web application tool for processing ChIP-chip and ChIP-seq data Bioinformatics 2011 27 3 428 430 21138948 10.1093/bioinformatics/btq669 PMC3031039

[B3] Ji H Jiang H Ma W Johnson DS Myers RM Wong WH An integrated software system for analyzing ChIP-chip and ChIP-seq data Nat Biotechnol 2008 26 11 1293 1300 18978777 10.1038/nbt.1505 PMC2596672

[B4] Alawi M Kurtz S Beckstette M CASSys: an integrated software-system for the interactive analysis of ChIP-seq data J Integr Bioinform 2011 8 2 155 21690655 10.2390/biecoll-jib-2011-155

[B5] Quinlan AR Hall IM BEDTools: a flexible suite of utilities for comparing genomic features Bioinformatics 2010 26 6 841 842 20110278 10.1093/bioinformatics/btq033 PMC2832824

[B6] Zhu LJ Gazin C Lawson ND Pages H Lin SM Lapointe DS Green MR ChIPpeakAnno: a Bioconductor package to annotate ChIP-seq and ChIP-chip data BMC Bioinformatics 2010 11 237 20459804 10.1186/1471-2105-11-237 PMC3098059

[B7] Strozzi F Aerts J A Ruby API to query the Ensembl database for genomic features Bioinformatics 2011 27 7 1013 1014 21278190 10.1093/bioinformatics/btr050 PMC3065687

[B8] Giardine B Riemer C Hardison RC Burhans R Elnitski L Shah P Zhang Y Blankenberg D Albert I Taylor J Miller W Kent WJ Nekrutenko A Galaxy: a platform for interactive large-scale genome analysis Genome Res 2005 15 10 1451 1455 16169926 10.1101/gr.4086505 PMC1240089

[B9] Karolchik D Hinrichs AS Furey TS Roskin KM Sugnet CW Haussler D Kent WJ The UCSC Table Browser data retrieval tool Nucleic Acids Res 2004 32 Database D493 496 14681465 10.1093/nar/gkh103 PMC308837

[B10] Muller H Zammataro L Bucci G AnnotateGenomicRegions: a web application EMBnetjournal 2012 18 Supplement B 135 137 10.1186/1471-2105-15-S1-S8 PMC4015944 24564446

[B11] Pruitt KD Tatusova T Maglott DR NCBI Reference Sequence (RefSeq): a curated non-redundant sequence database of genomes, transcripts and proteins Nucleic Acids Res 2005 33 Database D501 504 15608248 10.1093/nar/gki025 PMC539979

[B12] Flicek P Amode MR Barrell D Beal K Brent S Chen Y Clapham P Coates G Fairley S Fitzgerald S Gordon L Hendrix M Hourlier T Johnson N Kahari A Keefe D Keenan S Kinsella R Kokocinski F Kulesha E Larsson P Longden I McLaren W Overduin B Pritchard B Riat HS Rios D Ritchie GR Ruffier M Schuster M Ensembl 2011 Nucleic Acids Res 2011 39 Database D800 806 21045057 10.1093/nar/gkq1064 PMC3013672

[B13] Kent WJ Sugnet CW Furey TS Roskin KM Pringle TH Zahler AM Haussler D The human genome browser at UCSC Genome Res 2002 12 6 996 1006 12045153 10.1101/gr.229102 PMC186604

